# Changes in Coordination and Its Variability with an Increase in Functional Performance of the Lower Extremities

**DOI:** 10.3390/bios13020156

**Published:** 2023-01-19

**Authors:** Clint Hansen, Baraah Chebil, John Cockroft, Edoardo Bianchini, Robbin Romijnders, Walter Maetzler

**Affiliations:** 1 Neurogeriatrics, University Hospital Kiel, 24105 Kiel, Germany; 2 Neuromechanics Unit, Stellenbosch University, Stellenbosch 7602, South Africa; 3 NESMOS Department, Sapienza University of Rome, 00185 Rome, Italy; 4 Digital Signal Processing and System Theory, Kiel University, 24118 Kiel, Germany

**Keywords:** Short Physical Performance Battery, SPPB, wearables, kinematics, inter-joint coordination, cyclogram, neurogeriatrics

## Abstract

Clinical gait analysis has a long-standing tradition in biomechanics. However, the use of kinematic data or segment coordination has not been reported based on wearable sensors in “real-life” environments. In this work, the skeletal kinematics of 21 healthy and 24 neurogeriatric participants was collected in a magnetically disturbed environment with inertial measurement units (IMUs) using an accelerometer-based functional calibration method. The system consists of seven IMUs attached to the lower back, the thighs, the shanks, and the feet to acquire and process the raw sensor data. The Short Physical Performance Battery (SPPB) test was performed to relate joint kinematics and segment coordination to the overall SPPB score. Participants were then divided into three subgroups based on low (0–6), moderate (7–9), or high (10–12) SPPB scores. The main finding of this study is that most IMU-based parameters significantly correlated with the SPPB score and the parameters significantly differed between the SPPB subgroups. Lower limb range of motion and joint segment coordination correlated positively with the SPPB score, and the segment coordination variability correlated negatively. The results suggest that segment coordination impairments become more pronounced with a decreasing SPPB score, indicating that participants with low overall SPPB scores produce a peculiar inconsistent walking pattern to counteract lower extremity impairment in strength, balance, and mobility. Our findings confirm the usefulness of SPPB through objectively measured parameters, which may be relevant for the design of future studies and clinical routines.

## 1. Introduction

The ability to walk is a fundamental requirement for many everyday activities. Deficits in walking ability increase the risk of reduced quality of life as well as disability, particularly due to an increased risk of falling among older adults [1]. 

One clinical tool frequently used to evaluate gait and balance ability in older adults is the Short Physical Performance Battery (SPPB) test. SPPB combines measures of lower extremity strength, balance, and mobility and has been used to predict the risk of decline in activities of daily living [2], falls [3,4], hospitalization [5,6], and mortality [7] in a variety of patient populations. The overall SPPB score ranges from 0 to 12 points (worst performance to best performance) where a change of 1 point can be considered clinically significant [8]. Moreover, a 1-point increase signifies 14% lower odds of hospital readmission within 12 months in non-demented patients aged 65 years and older [6], and cardiac patients with a post-surgical SPPB score <11 have a 3.8-fold risk of unplanned 30-day hospital readmission [5], compared to those having a score of ≥11. It has also been shown in older adults undergoing transcatheter aortic valve replacement that if the SPPB score drops below 9, the probability of developing new disabilities in activities of daily life within the next year doubles [9]. Because of this diagnostic and predictive power, the SPPB has become a routine test for assessing mobility in many geriatric clinics and departments and is one of the most widely used tests in these patients worldwide.

However, the SPPB assessment does not currently consider any detailed biomechanical measures of walking ability, such as quantitative gait analysis, which may provide additional and clinically relevant information. This is particularly interesting because it potentially allows for more specific therapies to be offered, e.g., by allied health therapists. Such quantitative gait analysis is now available and in widespread use [10] although it is often still restricted to expensive laboratory equipment and highly supervised assessments. One alternative technology promising more mobile, user-friendly gait analysis is wearable sensing. Inertial measurement units (IMUs) are small body-mounted sensors containing accelerometers, gyroscopes, and magnetometers that can track 3D human movement at a very granular level. IMU systems can utilize sensor fusion algorithms [11,12] and biomechanical modeling techniques to extract kinematic [13,14] and spatiotemporal gait parameters [15,16]. 

A complex motion such as walking requires precise coordination of body segments to maintain a fluent gait pattern [17,18]. Therefore, changes in coordination reflect arrhythmic walking patterns that could suggest potential neurological or orthopedic causes. Quantifying the intralimb coordination (in- or out of phase) [19] between the proximal and distal body segments joined at a particular joint, e.g., thigh–shank coordination, may reveal entirely new information on walking capacity and gait control across different mobility disorders [20]. A change in intralimb coordination may also be a consequence of impaired sensory feedback [21], changes in cadence [22], or running-related overuse injuries [23]. However, it is to our best knowledge unknown to date if or how segment-coordination and coordination variability change with a decrease in lower extremity strength, balance, and mobility capacity. 

Therefore, this study aimed to determine, in adults with and without mobility impairment, if IMU-measured changes in segment coordination and coordination variability correlate with a gold-standard measure of mobility in older adults, the SPPB test. 

## 2. Materials and Methods

### Participants

In total, 45 participants took part in this study (28 women (age: 51.8 years ± 25.7 years; weight: 66.4 ± 21.1 kg; height: 1.66 ± 0.07 m) and 17 men (age: 60.8 years ± 21.1 years; weight: 86.5 ± 25.7 kg; height: 1.66 ± 0.07 m). All participants were either inpatients at the neurogeriatric ward of the Neurology Center at the University Hospital of Kiel or hospital personal (n = 21). Among the variety of diagnoses, the three main diagnoses were stroke (n = 14), Parkinson’s disease (n = 6), and polyneuropathy (n = 6).

The study was conducted according to the guidelines of the Declaration of Helsinki and approved by the Ethics Committee of Kiel University (D438/18) and all participants provided written informed consent before participation. Participants were excluded when their fall risk was determined to be too high (>2 falls in the previous week), corrected visual acuity was below 60%, they scored ≤5 points in the Montreal Cognitive Assessment (MoCA) test [24,25], had current or past chronic substance abuse (except nicotine), and were not able to perform at least one of the walking tasks.

All participants performed the SPPB test at the University Hospital Kiel. The SPPB assesses lower extremity physical function: 3 standing balance tests, a 4-meter walk at the usual pace, and 5 unassisted chair rises. Subgroups were divided a posteriori based on the SPPB score as follows: low (4–6 points), moderate (7–9), and high (10–12) SPPB scores [26].

**Figure 1:**
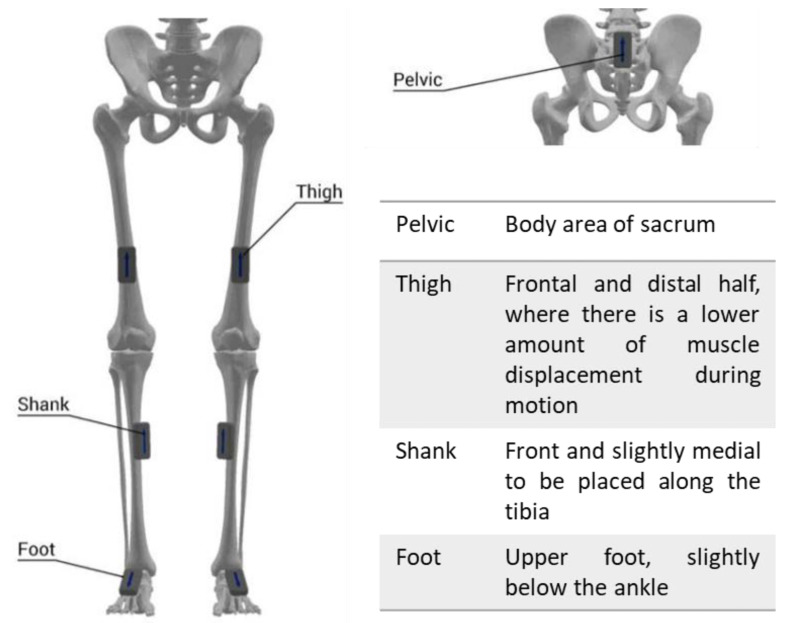
Placement of the inertial measurement units on the participants during the experimental procedure.

Participants were recorded while walking using an IMU motion capture system (Noraxon USA Inc., myoMOTION, Scottsdale, AZ, USA). Seven IMUs were attached to the body (pelvic, thighs, shanks, and feet) using elastic bands with a special housing for the IMU to clip into (Figure 1). The IMU data were collected at 200 Hz in the commercial software package (Noraxon MR3.16). 

In comparison to an optoelectronic motion capture system, the Noraxon IMU motion capture system shows valid and reliable results: After removing modeling offsets, differences for joint angles were <5°. Gait curves correlated highly between systems (r > 0.8), except hip rotation, pelvic tilt, and -obliquity. Within-session reliability of IMU-measured gait angles was clinically acceptable (standard error of measurement [SEM] <5°). Calibration poses were repeatable (SEM 0.3–2.2°). Pose accuracy revealed mean absolute differences (MAD) <5° for all angles except sagittal ankle, hip, and pelvis. IMU tracking accuracy demonstrated RMSE ≤ 2.0° [13,14]. Sensor-on-segment positions were obtained for the biomechanical model using a static standing (N-pose) calibration. Sensor-to-segment calibration of the biomechanical model was performed using a standard protocol prescribed by the supplier of the commercial system. Firstly, the patient stood in a static reference pose for 5 s (traditional standing neutral posture). Immediately after this, the subject walked forwards for 5–10 m, turned around and walked back to the original position on the floor, and assumed the original calibration posture and facing direction. The walking protocol compensates for the effects of magnetic interference on each sensor by exploiting the fact that the sensors mounted on the body segments move in the same direction over the floor. A sensor fusion algorithm was used to estimate the IMU orientation from the 3D accelerometers, gyroscopes, and magnetometers. Attitude is measured using an extension of Wiener filtering, whereas heading is measured with a dual-mode estimator that uses probability theory to identify magnetic distortion. The gyroscope bias is estimated using a third-order Kalman–Bucy filter [27]. 

## 3. Gait Assessment

Before the assessment of the SPPB, an acceleration-based functional calibration refinement procedure was applied to the commercial software to correct the effects of any magnetic field disturbances in the static pose calibration. The walking assessment task was performed at the preferred walking speed in a hospital corridor. Participants were asked to start walking once the examiner gave them a start sign and walked until the end of the 10 m walking distance, indicated by a colored tape on the corridor floor. 

## 4. IMU-Derived Variables

The IMU system automatically detects gait cycle events using an acceleration-based algorithm [13,14]. Each gait cycle of the protocol was extracted, and kinematic signals were segmented and resampled to 101 separate time points (0–100%) per gait cycle. Cadence, stride duration, stance, and swing phase duration as well as ranges of motion (ROM) for hip, knee, and ankle joint sagittal plane angles were calculated for approximately five gait cycles per participant. Intralimb coordination was assessed using indicators describing the shape and the consistency of cyclograms. The within-subject cycle-to-cycle consistency was quantified using the angular component of the coefficient of correspondence (ACC) [28]. The ACC denotes the degree of dispersion of the hip–knee cyclograms, i.e., the overall variability of the knee-hip relationship. In short, the ACC is computed based on the difference between consecutive frames $(l_{i})\;$ of the knee angle values $\left(x_{i}\right)$ and the hip angle values $\left(y_{i}\right)$, representing the change in the x and y directions of the cyclogram (Equation (1)). 

The angular direction of the line segment,$\;l_{i,i+1}$, between 2 consecutive points or frames is computed as:

(1) $$l_{i,i+1}=\sqrt{{\left(x_{i}-x_{i+1}\right)}^{2}+{\left(y_{i}-y_{i+1}\right)}^{2}}$$

The angular direction between the consecutive frames of $l_{i,i+1}$ is calculated using the sine and cosine.

(2) $$\sin\theta_{i,i+1}=\frac{y_{i,i+1}}{l_{i,i+1}}$$

(3) $$\cos\theta_{i,i+1}=\frac{x_{i,i+1}}{l_{i,i+1}}$$

The mean cosine ($\cos\bar{\theta }$) and sine ($\sin\bar{\theta }$) for a given frame-to-frame interval over multiple cycles was calculated, and the mean vector length for that frame-to-frame interval was then determined using Equation (4):

(4) $$a_{i,i+1}=\sqrt{(\cos{\bar{\theta }}_{i,i+1}{)}^{2}+(\sin{\bar{\theta }}_{i,i+1}{)}^{2}}$$

The arithmetic average, ACC, of all the mean vector lengths is found by Equation (5):

(5) $$\mathrm{ACC}=\frac{\left(a_{i,i+1}+a_{i+1,i+2}\ldots a_{n,n+1}\right)}{n}$$

For detailed explanations and equations please see [28]. The ACC ranges from 0 to 1 and the larger the value the less variable (i.e., less randomly distributed, more consistent) is the hip–knee relationship. If the relative motion between the hip and the knee is in perfect agreement over multiple cycles, then 1 indicates the maximal consistency between cycles. 

The shape difference between the cyclograms was quantified as the square root of the sum of squared distances (SSD) after uniform scaling and translation of the cyclogram centroids to the origin (see [20]).

(6) $$SSD_{i,i+1}=\sqrt{\sum_{i}{\left(x_{i}-x_{i+1}\right)}^{2}+{\left(y_{i}-y_{i+1}\right)}^{2}\;}$$

where $\left(x_{i}\right)$ represent the knee angle values and the hip angle values $\left(y_{i}\right)$, representing the change in the x and y directions.

Data analysis was performed using custom-written Matlab scripts (Matlab R2017a; The MathWorks Inc., Natick, MA, USA).

## 5. Statistical Analysis

JASP Team (2022) JASP (Version 0.16.1) was used for all statistical analyses. The normality of data distribution was tested using histograms and the Shapiro–Wilk test. Based on the sample size and the non-normally distributed values, variables were compared across the three subgroups using the Kruskal–Wallis test including the Dunn post hoc in case of a significant main effect. To assess the relationship between the IMU-derived variables and the overall SPPB scores non-parametric ρ (rho) Spearman correlation coefficient was calculated. To translate the correlation coefficient into descriptors the following cutoff points were chosen based on [29]: 0 to 0.3: negligible; >0.3 to 0.5: low; >0.5 to 0.7: moderate; >0.7 to 0.9: strong and >0.9 to 1: very strong. Measures are reported as mean ± SD for numerical variables or N (%) for categorical ones. To counteract the multiple comparisons problems, a Bonferroni correction was applied to the initial significance threshold of α < 0.05.

## 6. Results

The overall SPPB scores of the participants ranged from 4 to 12 points. The “low” group consisted of 8 participants (18%) (age: 79.5 ± 6.8 years; weight: 75.6 ± 9.8 kg; height: 1.69 ± 0.11 m), the “moderate” group of 14 participants (31%) (age: 74.8 ± 8.8 years; weight: 73.9 ± 21.4 kg; height: 1.69 ± 0.07 m) and the “high” group of 23 participants (51%) (age: 34.8 years ± 14.9 years; weight: 73.5 ± 17.0 kg; height: 1.73 ± 0.12 m). 

The overall SPPB score showed a strong positive correlation with hip and knee ROM, a moderate correlation with ankle ROM and cadence, and a low correlation with ACC. The score also demonstrated a strong negative correlation with stance duration, a moderate correlation with stride duration and SSD, and a low correlation with swing duration (Table 1).

**Table 1:** Correlation analysis of the IMU-derived parameters from the gait assessment with the SPPB and subgroup comparisons.

IMU-Derived Parameter	Correlation with SPPB Total Score		Sub-Group Comparison	
	ρ	*p*	H(2)	*p*
Stride duration	−0.690	<0.001 *	19.805	<0.001 *
Stance duration	−0.754	<0.001 *	24.071	<0.001 *
Swing duration	−0.362	0.015	5.761	0.056
Hip ROM	0.717	<0.001 *	25.918	<0.001 *
Knee ROM	0.741	<0.001 *	26.125	<0.001 *
Ankle ROM	0.537	0.001 *	13.591	<0.001 *
Cadence	0.678	<0.001 *	17.506	<0.001 *
SSD	−0.552	<0.001 *	14.057	<0.001 *
ACC	0.300	0.045	7.469	0.024

Subgroups were defined as low, moderate, and high according to the Short Physical Performance (SPPB) score. Correlations of the IMU-derived parameters were calculated using Spearman’s Rho test, subgroup comparison was performed using the Kruskal–Wallis test. ROM = Range of motion, ACC = angular component of coefficient of correspondence and SSD = square root of the sum of squared distances * Significant after Bonferroni correction.

**Figure 2:**
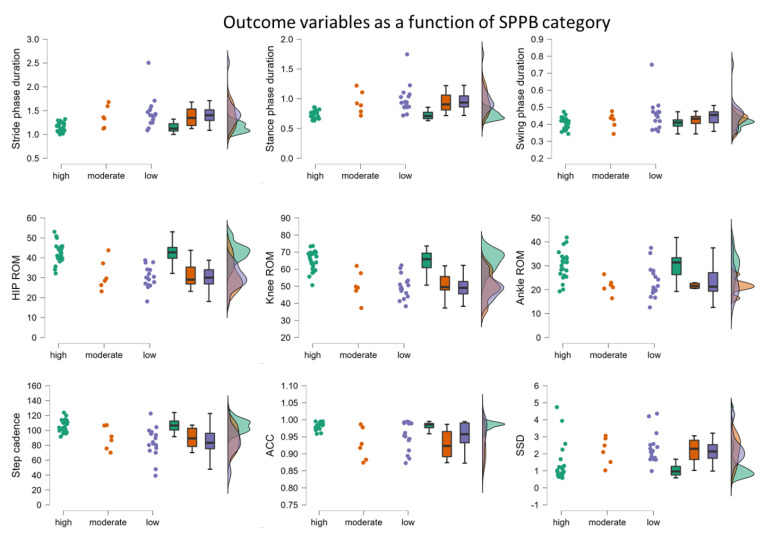
Raincloud plots (containing a violin + boxplot + jittered dataset combination) highlighting the individual data points, probability distribution, and statistical inference at a glance via medians and interquartile range for the nine extracted IMU-derived parameters as a function of SPPB categories: “low” (SPPB score range 4–6), “moderate” (7–9), and “high” (10–12).

Similarly, all IMU-derived parameters besides the swing phase and ACC duration were significantly different between the three SPPB subgroups (Table 1 and Figure 2). Pairwise comparisons showed that both the “moderate” and “low” groups were significantly different from the “high” SPPB group. However, there were no significant differences between the moderate and low groups (*p* > 0.05).

The raincloud plots highlight the variability across the three subgroups, with the “high” group showing the most consistent pattern and the least variability for the phase durations, hip and knee ROM, step cadence, and the within-subject cycle-to-cycle consistency (ACC). For the shape difference between the cyclograms (SSD) and the ankle ROM, the spread is relatively wide in all three subgroups, highlighting similarities between participants despite their (differing) SPPB scores.

**Figure 3:**
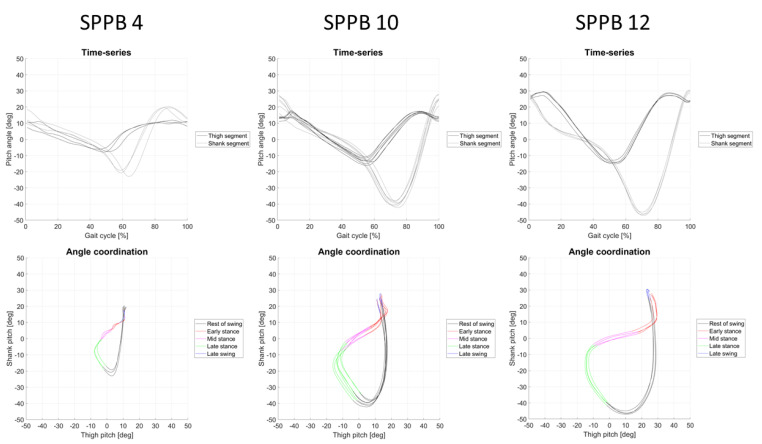
Joint motion of three exemplary participants is shown representing SPPB scores of 4, 10, and 12, respectively. The upper part of the figure shows the normalized joint angles of the hip and the knee, and the middle part shows the cyclograms of the hip–knee angles.

For visual interpretation of the potential differences between the SPPB scores and the IMU-derived parameters, three exemplary participants are shown representing SPPB scores of 4, 10, and 12, respectively (Figure 3). The SPPB 4 participant demonstrates a clear reduction in angle coordination between the thigh and shank segments in comparison to the SPPB 10 and 12 participants. 

## 7. Discussion

This study aimed to evaluate if changes in segment coordination and coordination variability correlate with the overall SPPB score in a population of 45 participants with strong divergent mobility. A wearable IMU system was used to acquire and process the raw sensor data. One walking assessment was performed to evaluate the relationship between cadence, stride duration, stance, and swing phase duration as well as hip, knee, and ankle joint ROM and joint coordination parameters cycle-to-cycle consistency (ACC) and shape differences (SSD) with changes in the overall SPPB score. The main finding of this study is that all IMU-derived parameters besides ACC and swing duration significantly correlated with the overall SPPB score. In addition, all IMU-derived parameters besides swing phase duration and ACC were significantly different between the low, moderate, and high SPPB subgroups, where the significances were mainly driven by the difference between the high SPPB group and the other two subgroups. 

Upon investigation of the individual IMU-derived parameters, participants with lower overall SPPB scores walked with shorter (less ROM) and slower (reduced cadence) steps, which indicates slower walking. While this could be expected, this study supports the findings with kinematic results obtained from an IMU system. The kinematic variables could be used to quantify the “quality of movement”, in contrast to the generally used spatiotemporal parameters. The quality assessment of movement has the potential to describe specific movement restrictions associated with neurological diseases. A change in the overall SPPB score cannot simply be translated into a change in cadence or ROM, but emphasizes that the SPPB is a predictive tool for possible disability and can aid in the monitoring of function in older people [30]. A reduction in ROM, as well as cadence, implies functional limitations of those participants which are coherent with the findings of [30,31], showing that the SPPB also allows for predicting the loss of ability to walk 400 m. 

The implication of functional limitations is supported by the ACC and SSD of the cyclograms. They suggest that impairments become more pronounced with a decreasing SPPB score. The increase in shape deformity and decrease in consistency did not occur randomly but rather happens as a function of SPPB worsening indicating that participants with low SPPB scores produce a peculiar walking pattern to counteract lower extremity strength, balance, and mobility. Participants with lower SPPB scores spent longer in stance, but not in swing, indicating that they increased the proportion of the gait cycle spent in double support. Increasing double limb support could be seen as compensation for deficits in balance and postural control [32]. This is supported by findings from [31] who showed that in comparison with non-fallers, fallers exhibited a longer stance phase and a shorter swing phase which could mean that they maintain gait stability by increasing the stance phase. In a study with healthy adults [33] consciously altered the double-support percentage of the gait cycle without changing walking speed. The gait alterations are shown in larger double-limb support times, reduced single support, and a decreased joint range of motion, underlining the current results. This could also mean that a prolonged double-support time was used to gain stability but also could be an indicator for a potential prolonged relaxation phase. It may be that the lower SPPB score group takes a slower walking speed into account while gaining time to increase relaxation due to an augmented arterial blood flow into the skeletal muscles during the relaxation phase [34]. 

In terms of clinical application, the use of cyclograms and inter-joint coordination metrics has been successfully applied both in sports and clinical applications. While running, the assessment of inter-joint coordination may help inform about injury prevention, risk of intervention-induced injury [22], or treatment strategies for less-experienced runners [35]. Having baseline measures of segment coordination could help with injury recovery monitoring and physiotherapy [36]. Segment coordination metrics allow us to distinguish between patient groups such as osteoarthritis and total hip arthroplasty [37] but also serve as a tool in the identification of specific movement coordination deficits in neurological impaired cohorts [38]. For example, in stroke patients, the coordination of body segments differs between preplanned and reactive changes in walking direction [39], highlighting its usefulness as a potential marker for disease progression (e.g., neuropathy) [40] or falls. A major cause of falls is incorrect weight transfer; thus, impairments in inter-joint coordination during the weight transfer might be an important risk factor for falls in older persons [41]. Parkinson’s disease patients show an increased variability between left and right arm swing [42], which may affect weight transfer and result in an increased risk of falls. 

It would be worthwhile to mention the strengths and limitations of the current study. We acknowledge the fact that the sample heterogeneity, especially the age differences between subgroups, may cover some of the underlying motor symptoms. However, the sample also represents the day-to-day patients seen on the neurogeriatric wards. The grouping of our participants into three subgroups also emphasizes the importance of the SPPB as the differences between the subgroups were larger than the minimally significant change estimates of 0.3–0.8 points and substantial changes of 0.4–1.5 points previously reported in older adults [31]. The SPPB is known for its ceiling effects and has limitations in the assessment of physical functioning across the full spectrum of community-dwelling adults [43]. Depending on the cohort the ceiling effect is important, and results need to be put into context. It has been shown that in 70–74-year-old Norwegians, 80% of the cohort had a SPPB score of >10, whereas [44] showed similar scores only in 40% of 70–79-year-old adults in Colombia and [4] only a rate of 45% in Italian adults aged over 65 years of age. As the investigated cohort had a large age range and also represents patients from the neurogeriatric wards, the ceiling effect is not likely to affect the outcomes of this study. 

With regard to the chosen parameters, they only represent a small number of potential candidates but providing a few understandable parameters is often more helpful than swamping the analysis with arbitrary parameters. The parameters extracted in this study are based on the raw data collected from the IMU positioned from the lower limbs. Consequently, we encourage the evaluation of other parameters that are extracted from individual IMU positions such as the feet, or even comparing multiple analysis algorithms, to understand how the algorithm choice influences the comparison with the SPPB. 

## 8. Conclusions

In this work, an IMU-derived inter-joint coordination calculation was proposed and tested to relate joint kinematics and segment coordination to the overall SPPB score. Our findings suggest that lower limb ROM and joint segment coordination decrease as a function of the SPPB score, whereas the segment coordination variability increases. This indicates that participants with low SPPB scores produce a peculiar inconsistent walking pattern to counteract lower extremity impairment in strength, balance, and mobility. These results underline the usefulness of SPPB through objectively measured parameters and may be relevant for the design of future studies and application in clinical routine. In addition, the acceleration-based functional calibration refinement procedure allowed for correcting the effects of any magnetic field disturbances, and the movement assessment was hence possible in the hospital corridor, meaning that the gait assessment is really not restricted to one laboratory but can be used directly on the clinical ward. 

## Data Availability

Up on reasonable request from correspondent author.

## References

[1] Afilalo J., Lauck S., Kim D.H., Lefèvre T., Piazza N., Lachapelle K., Martucci G., Lamy A., Labinaz M., Peterson M.D. (2017). Frailty in older adults undergoing aortic valve replacement: The FRAILTY-AVR study. J. Am. Coll. Cardiol..

[2] Ashikaga K., Saji M., Takanashi S., Nagayama M., Akashi Y.J., Isobe M. (2019). Physical performance as a predictor of midterm outcome after mitral valve surgery. Heart Vessel..

[3] Awai L., Curt A. (2014). Intralimb coordination as a sensitive indicator of motor-control impairment after spinal cord injury. Front. Hum. Neurosci..

[4] Bellettiere J., Lamonte M.J., Unkart J., Liles S., Laddu-Patel D., Manson J.E., Banack H., Seguin-Fowler R., Chavez P., Tinker L.F. (2020). Short Physical Performance Battery and Incident Cardiovascular Events Among Older Women. JAHA.

[5] Bergland A., Strand B.H. (2019). Norwegian reference values for the Short Physical Performance Battery (SPPB): The Tromsø Study. BMC Geriatr..

[6] Berner K., Cockcroft J., Louw Q. (2020). Kinematics and temporospatial parameters during gait from inertial motion capture in adults with and without HIV: A validity and reliability study. BioMed. Eng. OnLine.

[7] Berner K., Cockcroft J., Morris L.D., Louw Q. (2020). Concurrent validity and within-session reliability of gait kinematics measured using an inertial motion capture system with repeated calibration. J. Bodyw. Mov. Ther..

[8] Bernstein N. (1967). The Coordination and Regulation of Movements.

[9] Cirstea M.C., Mitnitski A.B., Feldman A.G., Levin M.F. (2003). Interjoint coordination dynamics during reaching in stroke. Exp. Brain Res..

[10] Crawley M.J. (2005). Statistics: An Introduction Using R..

[11] Del Din S., Elshehabi M., Galna B., Hobert M.A., Warmerdam E., Suenkel U., Brockmann K., Metzger F., Hansen C., Berg D. (2019). Gait analysis with wearables predicts conversion to parkinson disease. Ann. Neurol..

[12] Desai G.A., Gruber A.H. (2021). Segment coordination and variability among prospectively injured and uninjured runners. J. Sport. Sci..

[13] Dewolf A.H., Mesquita R.M., Willems P.A. (2020). Intra-limb and muscular coordination during walking on slopes. Eur. J. Appl. Physiol..

[14] Field-Fote E.C., Tepavac D. (2002). Improved intralimb coordination in people with incomplete spinal cord injury following training with body weight support and electrical stimulation. Phys. Ther..

[15] Geritz J., Maetzold S., Steffen M., Pilotto A., Corrà M.F., Moscovich M., Rizzetti M.C., Borroni B., Padovani A., Alpes A. (2020). Motor, cognitive and mobility deficits in 1000 geriatric patients: Protocol of a quantitative observational study before and after routine clinical geriatric treatment—The ComOn-study. BMC Geriatr..

[16] Ghahramani M., Mason B., Pearsall P., Spratford W. (2022). An Analysis of Lower Limb Coordination Variability in Unilateral Tasks in Healthy Adults: A Possible Prognostic Tool. Front. Bioeng. Biotechnol..

[17] Gommans L.N.M., Smid A.T., Scheltinga M.R.M., Cancrinus E., Brooijmans F.A.M., Meijer K., Teijink J.A. (2017). Prolonged stance phase during walking in intermittent claudication. J. Vasc. Surg..

[18] Hafer J.F., Peacock J., Zernicke R.F., Agresta C.E. (2019). Segment Coordination Variability Differs by Years of Running Experience. Med. Sci. Sport. Exerc..

[19] Hafer J.F., Silvernail J.F., Hillstrom H.J., Boyer K.A. (2016). Changes in coordination and its variability with an increase in running cadence. J. Sport. Sci..

[20] Hamill J., Palmer C., Van Emmerik R.E.A. (2012). Coordinative variability and overuse injury. Sport. Med. Arthrosc. Rehabil. Ther. Technol..

[21] Hollands K.L., van Vliet P., Zietz D., Wing A., Wright C., Hollands M.A. (2010). Stroke-related differences in axial body segment coordination during preplanned and reactive changes in walking direction. Exp. Brain Res..

[22] Ihlen E.A.F. (2014). Age-related changes in inter-joint coordination during walking. J. Appl. Physiol..

[23] Kwon S., Perera S., Pahor M., Katula J.A., King A.C., Groessl E.J., Studenski S.A. (2009). What is a meaningful change in physical performance? Findings from a clinical trial in older adults (the LIFE-P study). J. Nutr. Health Aging.

[24] Latash M.L. (1998). Progress in Motor Control.

[25] Lauretani F., Ticinesi A., Gionti L., Prati B., Nouvenne A., Tana C., Meschi T., Maggio M. (2019). Short-Physical Performance Battery (SPPB) score is associated with falls in older outpatients. Aging Clin. Exp. Res..

[26] Lin C.-C., Wagenaar R.C. (2018). The impact of walking speed on interlimb coordination in individuals with Parkinson’s disease. J. Phys. Ther. Sci..

[27] Longworth J.A., Chlosta S., Foucher K.C. (2018). Inter-joint coordination of kinematics and kinetics before and after total hip arthroplasty compared to asymptomatic subjects. J. Biomech..

[28] Morris M.E., Cantwell C., Vowels L., Dodd K. (2002). Changes in gait and fatigue from morning to afternoon in people with multiple sclerosis. J. Neurol. Neurosurg. Psychiatry.

[29] Nasreddine Z.S., Phillips N.A., Bédirian V., Charbonneau S., Whitehead V., Collin I., Cummings J.L., Chertkow H. (2005). The Montreal Cognitive Assessment, MoCA: A brief screening tool for mild cognitive impairment. J. Am. Geriatr. Soc..

[30] Nez A., Fradet L., Marin F., Monnet T., Lacouture P. (2018). Identification of noise covariance matrices to improve orientation estimation by kalman filter. Sensors.

[31] Nikolaus T. (2005). Gang, gleichgewicht und stürze—Ursachen und konsequenzen. Dtsch. Med. Wochenschr..

[32] Perera S., Mody S.H., Woodman R.C., Studenski S.A. (2006). Meaningful change and responsiveness in common physical performance measures in older adults. J. Am. Geriatr. Soc..

[33] Phan G.-H., Hansen C., Tommasino P., Hussain A., Formica D., Campolo D. (2020). A complementary filter design on se(3) to identify micro-motions during 3d motion tracking. Sensors.

[34] Ramírez-Vélez R., Pérez-Sousa M.A., Venegas-Sanabria L.C., Cano-Gutierrez C.A., Hernández-Quiñonez P.A., Rincón-Pabón D., García-Hermoso A., Zambom-Ferraresi F., de Asteasu M.S., Izquierdo M. (2020). Normative Values for the Short Physical Performance Battery (SPPB) and Their Association With Anthropometric Variables in Older Colombian Adults. The SABE Study, 2015. Front. Med..

[35] Rizun P.R. (2010). On the Estimation of Angular Orientation with Microelectromechanical Systems. Unpublished. Ph.D. Thesis.

[36] Sathananthan J., Lauck S., Piazza N., Martucci G., Kim D.H., Popma J.J., Asgar A.W., Perrault L.P., Lefèvre T., Labinaz M. (2019). Habitual Physical Activity in Older Adults Undergoing TAVR: Insights From the FRAILTY-AVR Study. JACC Cardiovasc. Interv..

[37] Schniepp R., Möhwald K., Wuehr M. (2019). Clinical and automated gait analysis in patients with vestibular, cerebellar, and functional gait disorders: Perspectives and limitations. J. Neurol..

[38] Vasunilashorn S., Coppin A.K., Patel K.V., Lauretani F., Ferrucci L., Bandinelli S., Guralnik J.M. (2009). Use of the Short Physical Performance Battery Score to predict loss of ability to walk 400 meters: Analysis from the InCHIANTI study. J. Gerontology. Ser. A Biol. Sci. Med. Sci..

[39] Veronese N., Bolzetta F., Toffanello E.D., Zambon S., De Rui M., Perissinotto E., Coin A., Corti M.-C., Baggio G., Crepaldi G. (2014). Association between Short Physical Performance Battery and falls in older people: The Progetto Veneto Anziani Study. Rejuvenation Res..

[40] Volpato S., Cavalieri M., Sioulis F., Guerra G., Maraldi C., Zuliani G., Fellin R., Guralnik J.M. (2011). Predictive value of the short physical performance battery following hospitalization in older patients. J. Gerontol. A Biol. Sci. Med. Sci..

[41] Wennie Huang W.N., Perera S., Vanswearingen J., Studenski S. (2010). Performance Measures Predict Onset of Activity of Daily Living Difficulty in Community-Dwelling Older Adults. J. Am. Geriatr. Soc..

[42] Williams D.S., Martin A.E. (2019). Gait modification when decreasing double support percentage. J. Biomech..

[43] Yi L.C., Sartor C.D., Souza F.T., Sacco IC N. (2016). Intralimb Coordination Patterns in Absent, Mild, and Severe Stages of Diabetic Neuropathy: Looking Beyond Kinematic Analysis of Gait Cycle. PLoS ONE.

[44] Zijlstra W., Hof A.L. (2003). Assessment of spatio-temporal gait parameters from trunk accelerations during human walking. Gait Posture.

